# Scalable preparation of alternating block copolymer particles with inverse bicontinuous mesophases

**DOI:** 10.1038/s41467-019-09324-5

**Published:** 2019-03-27

**Authors:** Fei Lv, Zesheng An, Peiyi Wu

**Affiliations:** 1 State Key Laboratory of Macromolecular Engineering of Polymers, Department of Macromolecular Science and Laboratory of Advanced Materials, Fudan University, Shanghai, 200433 China; 2 Institute of Nanochemistry and Nanobiology, College of Environmental and Chemical Engineering, Shanghai University, Shanghai, 200444 China

## Abstract

Block copolymer particles with controlled morphologies are of great significance in nanomaterials and nanotechnology. However, ordered inverse morphologies are difficult to achieve due to complex mechanism and formation conditions. Here we report scalable preparation of amphiphilic alternating block copolymer particles with inverse bicontinuous mesophases via polymerization-induced self-assembly (PISA). Concentrated dispersion copolymerizations (up to 40% solid content) of styrene (St) and pentafluorostyrene (PFS) employing a short poly(*N,N*-dimethylacrylamide) (PDMA_29_) stabilizer block lead to the formation of well-defined, highly asymmetric PDMA_29_-*b*-P(St-*alt*-PFS)_x_ block copolymers with precise compositions and various morphologies, from simple spheres to ordered inverse cubosome mesophases. The particle morphology is affected by the molecular weight, solid content, and nature of the cosolvents. The cubosome structure is confirmed by electron microscopies and small angle X-ray scattering spectroscopy. This scalable PISA approach offers facile access to ordered inverse mesophases, significantly expanding the PISA morphology scope and enabling its applicability to the materials science fields.

## Abstract (web summary)

Block copolymer particles with controlled morphologies play a vital role in materials science and nanotechnology. Here the authors show a scalable preparation of amphiphilic alternating block polymer particles with inverse bicontinuous mesophases via polymerization-induced self-assembly.

## Introduction

Block copolymer (BCP) particles with controlled morphologies play a vital role in materials science and nanotechnology^1–8^. BCP self-assembly has produced various morphologies such as spheres, cylinders, vesicles, bicontinuous, and other multigeometry structures. The particle morphology of amorphous BCPs is determined by the relative block ratio and interfacial area according to the packing parameter *p* = *V/a*_0_*l*_c_ (*V* the volume of the core-forming block, *a*_0_ the interfacial area, and *l*_c_ the length of the core-forming block)^9^. While packing parameter reflects the geometry of the particles, many parameters have been demonstrated to influence the morphology, including the solubility parameter^10,11^, the incompatibility between BCPs^12^, topology^13–15^, and others^16^. BCP particles have been widely produced using solution self-assembly approach that has recognized limitations of multistep processing and low concentration^17,18^. In contrast, polymerization-induced self-assembly (PISA)^19–23^, combining simultaneous polymerization and in situ self-assembly, enables efficient and reliable access to various morphologies at high concentration (≥10% solid content). PISA has been explored to prepare a wide range of morphologies using various types of controlled/living polymerization techniques^13,24–46^. Despite rapid progress in this emergent field, current PISA research has predominantly focused on morphologies with *p* ≤ 1 (e.g., sphere, cylinder, and vesicle); in contrast, inverse morphologies with *p* > 1 have been rarely reported^47^.

Traditionally, ordered inverse morphologies have been difficult to achieve due to limited understanding of morphology transition mechanism, complex assembling procedures, and varying conditions, although hexasome was observed by Eisenberg and co-workers^48^ more than 20 years ago. Recently, much progress has been made with cubosomes and hexasomes having been produced via solution self-assembly of a few BCPs^49–55^. For instance, Kim and colleagues^56–58^ reported the formation of inverse bicontinuous cubic structures by dendritic-linear block copolymers with a dendritic hydrophilic block. These mesoscale particles are very attractive for applications in templating, separation, and adsorption and as bioreactors due to their highly porous structure and high surface area^51,56,58^. In principle, the high concentration and robust nature of PISA would enable more facile generation of such inverse morphologies that require aggregation/fusion of intermediate particles and the continuous changing of the surface curvature. Unfortunately, there exits only one literature report that claimed the formation of hexasome via PISA of polystyrene-based BCPs^47^. The paucity of reported examples of inverse morphologies limits the versatility of PISA. Thus, it is highly desirable to capitalize on the merits of PISA to prepare inverse morphologies to further expand its scope and utility.

Herein, we report the preparation of inverse mesophases comprising amphiphilic alternating BCPs via dispersion copolymerization^59^. A detailed and most extended morphological transition sequence is revealed starting from simple spheres all the way to complex inverse mesophases, i.e., cubosomes.

The clearly defined cubosomes obtained via PISA are confirmed by transmission electron microscopy (TEM), scanning electron microscopy (SEM), and small-angle X-ray scattering (SAXS) studies.

## Results

### Synthesis of PDMA-*b*-P(St-*alt*-PFS) alternating BCPs

**Fig. 1:**
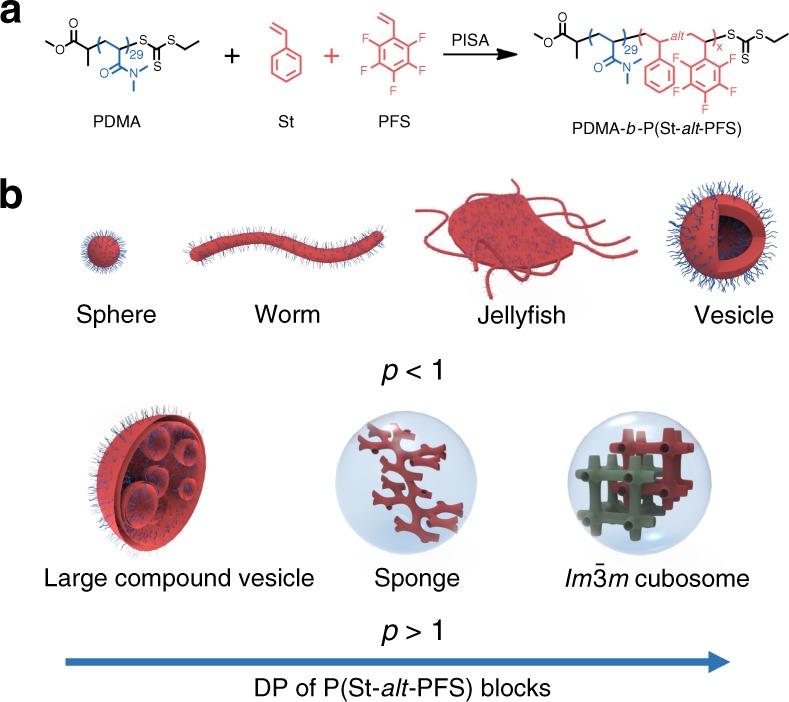
Preparation of PDMA-*b*-P(St-*alt*-PFS) block copolymer (BCP) particles with inverse mesophases via polymerization-induced self-assembly (PISA). **a** Synthetic scheme of PDMA-*b*-P(St-*alt*-PFS) alternating block copolymers. **b** Schematic illustration of morphological evolution of PDMA-*b*-P(St-*alt*-PFS) particles with increasing degree of polymerization of the P(St-*alt*-PFS) block (the lattice diagrams show bicontinuous internal structures of the nano-objects investigated in this study. For clarity, the bilayers that surround the solvent channels are drawn. The green- and red-colored regions indicate two non-intersecting networks of solvent channels within the bicontinuous structures). PDMA poly(*N,N*-dimethylacrylamide), St styrene, PFS pentafluorostyrene

As depicted in Fig. 1, reversible addition-fragmentation chain transfer (RAFT) dispersion copolymerizations of electron-rich styrene (St) with electron-deficient pentafluorostyrene (PFS) are conducted using a solvophilic poly(*N,N*-dimethylacrylamide) (PDMA) stabilizer block to afford various BCP particles. Copolymerization of St with PFS is known to generate highly alternating copolymers^60,61^. Thus, this PISA approach generates PDMA-*b*-P(St-*alt*-PFS) particles comprising a solvophilic PDMA block and a solvophobic alternating P(St-*alt*-PFS) block. Previously, Pan and colleagues^47^ claimed the formation of hexasome via PISA, but the monomer conversion was substantially incomplete (≤12.5%) and an unambiguous assignment of hexasome was not demonstrated. In contrast, high conversions (typically ≥90%) are achieved in this work, and most significantly, the structure of the inverse mesophases accessed at a solid content as high as 40% is fully characterized.

Previous work on alternating copolymerization of St and PFS was conducted in homogeneous solution. In this work, the alternating tendency of St and PFS in dispersion copolymerization conducted in ethanol was confirmed by the reactivity ratios (*r*_St_ = 0.52, *r*_PFS_ = 0.16, *r*_St_*r*_PFS_ = 0.08) and equal consumption of these two monomers when polymerized at a 1:1 monomer feed ratio (Supplementary Table 1 and Supplementary Figs. 3 and 4).

### Morphological evolution of BCP particles in ethanol

In designing experimental conditions to access inverse morphologies, we recognized that highly asymmetric BCPs and high concentrations are necessary to drive a multiple-staged morphological transition. Thus, dispersion copolymerizations of St and PFS were investigated using a short solvophilic PDMA_29_ (*M*_n_ = 3.1 kg mol^−1^, *Đ* = 1.10) and targeting degrees of polymerization (DPs) up to 600 for the solvophobic block at a high solid content (30%) in pure ethanol at 70 °C. The dispersion copolymerizations were analyzed by ^1^H nuclear magnetic resonance (NMR) spectroscopy and gel permeation chromatography (GPC) (Supplementary Table 2 and Supplementary Figs. 5–7). ^1^H NMR spectroscopy analysis indicated high conversions (≥90%) were achieved in most syntheses, and pseudo first-order kinetics was obtained by monitoring the dispersion copolymerization periodically. As measured by GPC, the molecular weight of PDMA_29_-*b*-P(St-*alt*-PFS)_x_ BCPs increased linearly with DP, and the molecular weight distribution was narrow (*Đ* ≤ 1.21). All these features confirm that controlled RAFT copolymerization was present under the dispersion conditions.

**Fig. 2:**
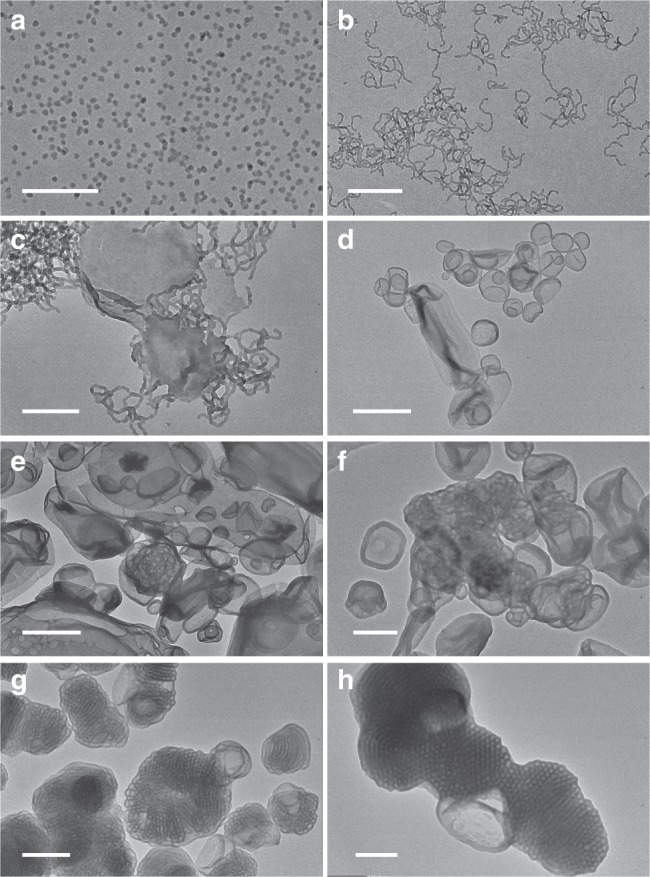
Transmission electron microscopy (TEM) micrographs of PDMA_29_-*b*-P(St-*alt*-PFS)_x_ block copolymer (BCP) particles synthesized in ethanol at 30% w/v and 70 °C. **a** *x* = 89 (scale bar: 200 nm), **b** *x* = 108 (scale bar: 500 nm), **c** *x* = 123 (scale bar: 200 nm), **d** *x* = 191 (scale bar: 1 μm), **e** *x* = 270 (scale bar: 500 nm), **f** *x* = 342 (scale bar: 500 nm), **g** *x* = 356 (scale bar: 1 μm), **h** *x* = 450 (scale bar: 500 nm). PDMA poly(*N,N*-dimethylacrylamide), St styrene, PFS pentafluorostyrene

The morphology of the PDMA_29_-*b*-P(St-*alt*-PFS)_x_ particles was studied by TEM. As shown in Fig. 2, the morphology for this series of BCP particles is extremely rich and evolves from simple spheres finally to complex inverse mesophases as the DP of the solvophobic alternating copolymer block increases. Uniform spheres with a diameter of 19 ± 2 nm (Fig. 2a) were obtained at a low DP (*x* = 89). Fusion of these spheres led to the formation of pure worms with a similar diameter but a polydisperse length (up to several micrometers) when *x* increased to 108 (Fig. 2b), consistent with the formation of a free-standing gel of the corresponding particle dispersion (Supplementary Fig. 8)^31,43^. Further increasing *x* promoted the formation of jellyfish-like intermediate morphologies (*x* = 123) via fusion of the worms (Fig. 2c), which further enclosed to form polydisperse vesicles with a membrane thickness of 24.6 ± 4.4 nm at *x* = 191 (Fig. 2d). Up to this stage, the morphological transition of PDMA_29_-*b*-P(St-*alt*-PFS)_x_ particles is consistent with the previously observed sphere-to-worm-to-vesicle sequence for amorphous BCPs^42^. As the DP of the P(St-*alt*-PFS) block continued to increase, some interesting morphologies with *p* > 1 became evident. Just like simple spheres, vesicles could also aggregate via inelastic collision, with large compound vesicles (LCVs) starting to form at *x* = 270 (Fig. 2e). Some ill-defined bicontinuous structures were observable due to further domain rearrangement of LCVs (Fig. 2f) and, remarkably, sponge-like bicontinuous structures fully matured when *x* reached 356 (Fig. 2g), although the internal structure still had a low regularity. Finally, particles (*D*_n_ ~1 µm) with an ordered internal structure of intertwined channels clearly formed at a high *x* value of 450, and this structure was assigned to cubosome from analysis by SAXS (vide infra). The dark channels (29 ± 7 nm) correspond to the solvophobic P(St-*alt*-PFS) domain, and the bright channels (32 ± 7 nm) correspond to the hollow space (or solvent for particle dispersions) surrounded by the solvophilic PDMA chains. SEM showed that some of the particles had a swirled curvature due to the alignment of some channels along the particle surface (Supplementary Fig. 9). The sphere→worm→vesicle→LCV→sponge→cubosome morphological evolution, with the successful capture of several intermediate morphologies such as jellyfish and ill-defined bicontinuous structures, represents the most extended morphological transition sequence observed in PISA and provides important insights into the morphological transition mechanism for amorphous BCPs.

### Effect of key parameters on morphology

To understand the effect of key parameters leading to the formation of cubosome, the total solid content and molecular weight of PDMA stabilizer block were adjusted, and the resulting morphologies were studied by TEM. When the total solid content was varied from 20 to 40%, inverse bicontinuous mesophases were only observed at a solid content ≥30% (Supplementary Table 3 and Supplementary Fig. 10). When a longer PDMA_48_ was used, only spheres were obtained at similar block ratios (Supplementary Table 4 and Supplementary Fig. 11). These results suggest that morphological transition from spheres to cubosomes requires effective inelastic collisions of particles to promote their fusion and chain/domain rearrangement, and a relative short stabilizer block and high solid content can facilitate such a process.

**Fig. 3:**
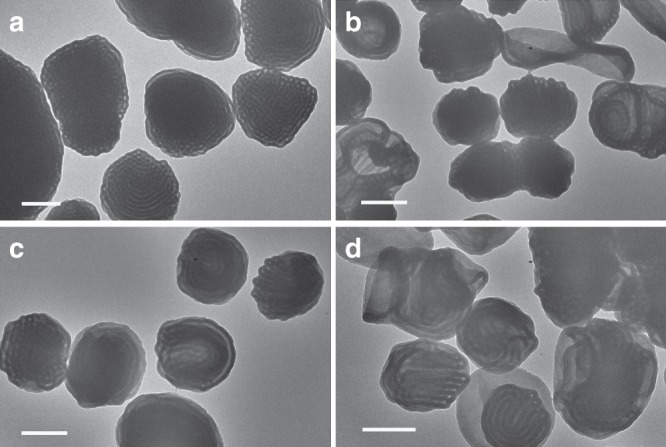
Transmission electron microscopy (TEM) micrographs of PDMA_29_-*b*-P(St-*alt*-PFS)_x_ block copolymer (BCP) particles synthesized at 30% w/v and 70 °C using 2% cosolvents. **a** Toluene, *x* = 465, **b** tetrahydrofuran (THF), *x* = 455, **c** *N,N*-dimethylformamide (DMF), *x* = 405, **d** dioxane, *x* = 450 (all scale bars: 500 nm). PDMA poly(*N,N*-dimethylacrylamide), St styrene, PFS pentafluorostyrene

Despite cubosomes can be successfully prepared via dispersion copolymerization of St and PFS in ethanol, a closer inspection of the particle shape reveals that the cubosome particles are not well dispersed. Some cubosome particles adhere to each other forming particle clusters. In order to improve the colloidal dispersity of cubosome particles, we next investigated the effect of cosolvent quality. To do this, 2% ethanol was replaced by different cosolvents including toluene, tetrahydrofuran (THF), *N,N*-dimethylformamide (DMF), and dioxane, and the dispersion copolymerizations were conducted at 30% solid content targeting DP 500 (Supplementary Table 5). As shown in Fig. 3, the internal structures of the particles produced using all cosolvents appear to have inverse bicontinuous structures, but the use of 2% toluene afforded a more ordered mesophase and a simultaneously improved colloidal dispersity as most of the particles are discrete entities rather than clusters, possibly attributable to enhanced solvation of the core-forming block. Thus, toluene was selected as an ideal cosolvent for further studies.

### Preparation of well-defined cubosomes

**Fig. 4:**
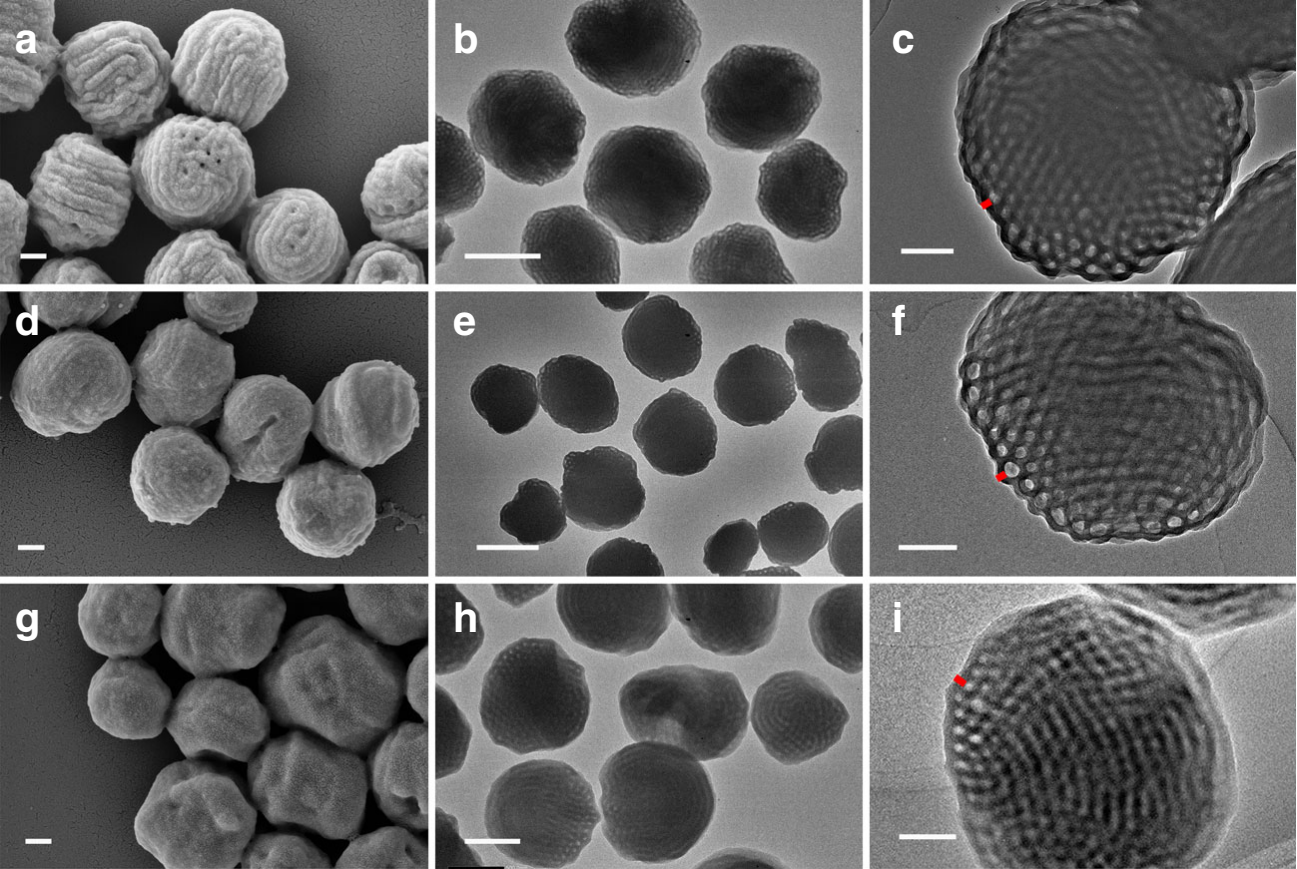
Scanning electron microscopy (SEM) and transmission electron microscopy (TEM) micrographs of PDMA_29_-*b*-P(St-*alt*-PFS)_x_ block copolymer (BCP) particles synthesized in 2% toluene/ethanol at 30% w/v and 70 °C. **a**–**c** *x* = 428 (scale bars: 200 nm, 500 nm and 200 nm), **d**–**f** *x* = 490 (scale bars: 200 nm, 1 μm and 200 nm), **g**–**i** *x* = 582 (scale bars: 200 nm, 500 nm and 200 nm). PDMA poly(*N,N*-dimethylacrylamide), St styrene, PFS pentafluorostyrene

Next, dispersion copolymerizations were further investigated at 30% solid content using different amounts of toluene as the cosolvent over a DP range similar to that in pure ethanol. When 2% toluene/ethanol was used, well-defined PDMA_29_-*b*-P(St-*alt*-PFS)_x_ block copolymers with *Đ* ≤ 1.30 up to a target DP of 600 were again synthesized with high conversions and controlled molecular weights (Supplementary Table 6 and Supplementary Figs. 12 and 13). The whole morphological transition sequence observed in pure ethanol was essentially reproduced in 2% toluene/ethanol (Supplementary Fig. 14). Remarkably, well-dispersed particles with inverse mesophases were obtained for DPs larger than 400, and their SEM and TEM micrographs are shown in Fig. 4. From the SEM micrographs, we can see these large particles with diameters around 1 µm are characterized by rough surfaces. The samples with core-forming block DPs of 428 and 490 have swirled patterns on the surfaces, while the sample with a DP of 582 has a certain faceted surface. These characteristic swirling patterns are formed due to the alignment of the bicontinuous channels on the surface with grooves being produced between neighboring channels. When DP is further increased, the internal morphology becomes more ordered and the different internal domains assume certain orientations and thus cause the formation of a faceted surface. Unlike typical spherical polymer particles with relatively smooth surfaces, the distinct surface patterns of these large particles reflect their ordered internal structures, which were probed by high-resolution TEM. As with the cubosome particles synthesized in pure ethanol, all the three samples synthesized in 2% toluene/ethanol have ordered bicontinuous internal structures, which are enclosed within a bilayer envelope with a thickness of 26–35 nm. The former two samples show similar internal patterns with similar channel size features: dark solvophobic channel diameter of 25 nm and hollow solvophilic channel diameter of 34 nm. However, the third sample appears to have different structural domains as well as different channel diameters; the dark solvophobic channel diameter is 35 nm and hollow solvophilic channel diameter is 22 nm.

**Fig. 5:**
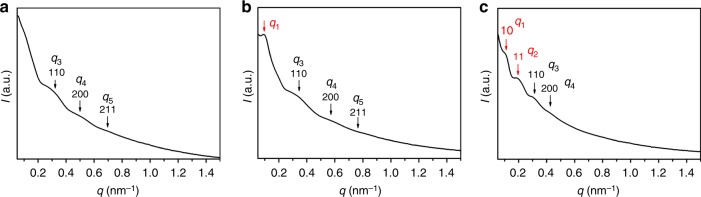
Small-angle X-ray scattering (SAXS) patterns of PDMA_29_-*b*-P(St-*alt*-PFS)_x_ block copolymer (BCP) particles. **a** *x* = 428, **b** *x* = 490, **c** *x* = 582. PDMA poly(*N,N*-dimethylacrylamide), St styrene, PFS pentafluorostyrene

The mesophases of these three samples were further studied by SAXS (Fig. 5). PDMA_29_-*b*-P(St-*alt*-PFS)_428_ shows three resolved peaks, which can be indexed to the 110, 200, and 211 planes of an *Im*3̄*m* cubosome structure with a lattice parameter *a* = 30 nm. PDMA_29_-*b*-P(St-*alt*-PFS)_490_ also shows the same set of three *Im*3̄*m* cubosome reflections (*a* = 27 nm), albeit at slightly larger *q* values, but another peak appears at a low *q*_1_ (0.092 nm^−1^). Although the exact structure associated with this *q*_1_ peak could not be deduced for this specific sample, it suggests that as the DP increases, a second structure, in addition to *Im*3̄*m*, emerges but in a minor fraction. Interestingly, this structure further evolves in PDMA_29_-*b*-P(St-*alt*-PFS)_582_, and the two peaks at low *q* can now be clearly assigned to the 10 and 11 reflections of a *p*6*mm* hexasome (*a* = 73 nm), while the 110 and 200 reflections of the *Im*3̄*m* cubosome (*a* = 30 nm) structure still remains. The SAXS data corroborate well with the TEM observation that the PDMA_29_-*b*-P(St-*alt*-PFS)_582_ particles have different internal domains, which have mixed *Im*3̄*m* and *p*6*mm* symmetries. On the basis of these results, we reasoned that if the DP of the core-forming block further increases, the particle morphology might transit from the mixed phase to pure *p*6*mm* hexasome. To explore this possibility, we conducted further experiments with DPs up to 800 at 30% solid content as well as increasing solid content to 40% at a fixed DP of 600 until the dispersions became colloidally unstable. However, the mixed phase in these particles remained unchanged (Supplementary Fig. 15). Further increasing toluene content (Supplementary Tables 7 and 8 and Supplementary Figs. 16, 17, 19 and 20) was also attempted, which, however, only resulted in the formation of less defined particles having less ordered internal structures (Supplementary Fig. 18 and Supplementary Fig. 21). These results indicate cubosome is the upper limit for the pure accessible morphology of this specific formulation under the investigated experimental conditions.

## Discussion

**Fig. 6:**
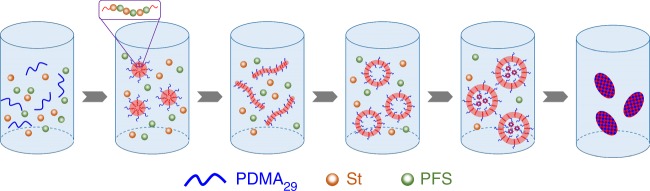
Schematic illustration of the polymerization-induced self-assembly (PISA) process of PDMA-*b*-P(St-*alt*-PFS) in toluene/ethanol with the increasing monomer conversions (for clarity, the earth yellow dots and green dots stand for the monomer styrene (St) and pentafluorostyrene (PFS), respectively. The blue lines stand for PDMA_29_ block and the red lines stand for P(St-*alt*-PFS) block. Moreover, the light red- and blue-colored regions stand for solvophobic and solvophilic regions in the particles). PDMA poly(*N,N*-dimethylacrylamide)

An efficient and scalable PISA approach is demonstrated for the preparation of BCP particles with inverse mesophases, with the observation of an extended morphological transition sequence from initial spheres to final inverse mesophases as the DP of the core-forming block increases (Fig. 6). The length of the stabilizer block length and the solid content are key parameters that affect the particle morphology transition. To achieve inverse mesophases such as cubosomes, the BCP particles need to experience a sequential morphological evolution from spheres to worms to vesicles to LCVs to sponge-like particles and finally to cubosomes. A suitably short stabilizer block is necessary to achieve colloidal stability and at the same time facilitate inelastic collision between particles. High solid content can enhance the population of inelastic collision and thus promotes the formation of higher-order morphologies. On the contrary, strong steric repulsion resulted from the presence of long stabilizer blocks on the particle surface effectively reduces the population of inelastic collision, leading to the formation of kinetically trapped spheres. The continuous morphological transition during dispersion copolymerization requires high chain mobility. Both the solvent and monomer can solvate/plasticize the growing polymer chains. However, as high monomer conversion is reached, the effect of monomer solvation becomes diminished. Thus, at the late stage of morphological transition, solvation from solvent becomes dominant. The solvent or dispersant (in this work ethanol) in dispersion polymerization is so chosen that it is a poor solvent for the core-forming block. In this regard, it is necessary to add a small fraction of a cosolvent (a good solvent for the core-forming block) (e.g., toluene used in this work) that can act as a plasticizer at the final stage of the polymerization or morphological transition. It should be emphasized that the amount of cosolvent should be kept minimal in order to avoid issues such as deformation of the shape of particles and weakening the driving force for self-assembly. By strategically adjusting these parameters and targeting highly asymmetric BCPs, pure cubosomes (as judged by the single set of SAXS pattern shown in Fig. 5a) can be accessed via PISA at high solid content. Given the high concentration and the continuous chain growth and reorganization as the unique driving force, PISA may become a practical and general approach for the preparation of inverse mesophases.

## Methods

### Materials

*N,N*-Dimethylacrylamide (DMA, 99%) and 2,3,4,5,6-pentafluorostyrene (PFS, 97%) were purchased from J&K Scientific. 2,2′-Azobis(2-methylpropionitrile) (AIBN, 99%) was purchased from Sigma-Aldrich. Styrene (St, AR), potassium phosphate tribasic trihydrate (K_3_PO_4_^.^3H_2_O, AR), dichloromethane (DCM, AR), ethanol (AR), *N,N*-dimethylformamide (DMF, AR), toluene (AR), tetrahydrofuran (THF, AR), 1,4-dioxane (AR), and acetone (AR) were obtained from Sinopharm Chemical Reagent. Ethanethiol (98%), carbon disulfide (99%), and methyl 2-bromopropionate (98%) were purchased from Energy Chemical. AIBN was recrystallized from ethanol prior to use. All monomers were purified by passing through an alkaline aluminum oxide column to remove the inhibitor. Other reagents were used without further purification.

### Synthesis of chain transfer agent

2-Ethylsulfanylthiocarbonylsulfanylpropionic acid methyl ester^62^ was synthesized according to the procedure below. In brief, ethanethiol (5.8 mL, 0.0805 mol) and K_3_PO_4_·3H_2_O (21.43 g, 0.0805 mol) were dissolved in 100 mL acetone and stirred at 0 °C for 30 min. Carbon disulfide (9.75 mL, 0.161 mol) was added dropwise slowly into the above solution. Then, the solution was heated to room temperature and stirred for 1.5 h. Methyl 2-bromopropionate (8.98 mL, 0.0805 mol) was added to the solution slowly and stirring was continued for 12 h. After the reaction, the solution was filtered and evaporated. Then, the crude product was dissolved in 50 mL DCM and washed three times with water. After being dried with anhydrous Na_2_SO_4_, pure product (10 g, yield 55%) was obtained by column chromatography. ^1^H NMR (400 MHz, CDCl_3_): δ [ppm] = 4.83 (q, 1 H), 3.73 (s, 3 H), 3.35 (q, 2 H), 1.59 (d, 3 H), 1.34 (t, 3 H).

### Synthesis of PDMA macro-CTAs

PDMA macro-chain transfer agents (CTAs) were synthesized according to a reported procedure^62^. Briefly, DMA (50 g, 0.5040 mol), AIBN (47.33 mg, 0.2880 mmol), and 2-ethylsulfanylthiocarbonylsulfanylpropionic acid methyl ester (3.23 g, 0.0144 mol) were dissolved in 100 mL DMF. The solution was heated to 70 °C after it was degassed with nitrogen for 30 min in an ice/water bath. The reaction was stopped after 2 h and the monomer conversion was determined to be 85% according to ^1^H NMR spectroscopy analysis. The solution was dialysis against ethanol and isolated by evaporation of ethanol to give the PDMA_29_ macro-CTA. ^1^H NMR (400 MHz, CDCl_3_): δ [ppm] = 3.70 (s, 3 H, H_g_), 3.34 (q, 2 H, H_b_), 2.75–3.20 (m, H_h_), 2.66 (br, H_c_), 1.26–1.89 (br, H_d_, H_f_), 1.22 (t, 3 H, H_a_), 1.11 (d, 3 H, H_e_). *M*_n,theory_ = 3.1 kg mol^−1^, *M*_n,GPC_ = 3.1 kg mol^−1^, and *Đ* = 1.10. PDMA_48_ macro-CTA was prepared according to the same procedure. *M*_n,theory_ = 5.0 kg mol^−1^, *M*_n,GPC_ = 5.1 kg mol^−1^, and *Đ* = 1.07 (Supplementary Fig. 1).

### Synthesis of PDMA_29_-*b*-P(St-*alt*-PFS)_x_ BCP particles

PDMA_29_-*b*-P(St-*alt*-PFS)_x_ block copolymer particles were synthesized by RAFT ethanolic dispersion copolymerization of St and PFS. The molar ratio of St to PFS was kept to be 1. The total solid content was 30% w/v. A typical procedure is described as follows: PDMA_29_ (44.4 mg, 0.0143 mmol), St (194.0 mg, 1.86 mmol), PFS (361.1 mg, 1.86 mmol), and AIBN (58 μL, 0.0123 g mL^−1^ in ethanol, 4.29 μmol) were dissolved in ethanol (2 mL). The reaction solution was degassed with N_2_ for 15 min in an ice/water bath and then heated to 70 °C for 60 h. To obtain block copolymers for characterization, more ethanol (5 mL) was added to the reaction solution (1 mL), followed by centrifugation (8000 rpm, 10 min) to collect the copolymers, which were washed three times with ethanol to give the pure copolymers. After vacuum drying at 40 °C for 12 h, the block copolymers were used for ^1^H NMR, ^19^F NMR, and GPC analysis (Supplementary Fig. 2). In most synthesis, the cosolvent (DMF, toluene, THF, 1,4-dioxane) was used as an internal standard. Comparing the peaks of internal standard with the double bond of St from ^1^H NMR, the conversions of monomers were calculated. For pure ethanolic PISA, the signal of the aromatic ring (6.82–7.21 ppm or 6.31–6.79 ppm) and the signal of methylene proton of PDMA_29_ (0.82–1.41 ppm) were used to calculate the DP of St. Comparing the DP with feed ratio of St, the conversions of monomers were obtained. ^1^H NMR (400 MHz, CDCl_3_): δ [ppm] = 6.82–7.21 (br, H_e_, H_f_), 6.31–6.79 (br, Hg), 2.78–3.19 (H_h_), 2.11–2.78 (br, H_a_, H_c_, H_i_), 1.45–2.11 (H_b_, H_d_), 0.82–1.41 (H_j_). ^19^F NMR (376 MHz, CDCl_3_): δ [ppm] = −142 (F_a_), −157 (F_c_), −163 (F_b_).

### Determination of reactivity ratios of St and PFS in ethanol

Copolymerization of St and PFS was performed using St/PFS feed molar ratios from 20:80 to 80:20. For instance, at a St/PFS = 20/80, a solution of St (90.97 mg, 0.68 mmol), PFS (529.03 g, 2.73 mmol), AIBN (89 μL of ethanolic solution, 0.019 g mL^−1^), and DMF (20 μL, internal standard) were dissolved in ethanol (2 mL). The total solid content was 30% w/v. The reaction solution was degassed with N2 for 15 min in an ice/water bath and then heated to 70 °C. A small aliquot was taken for conversion determination. A time of 10 min was sufficient to yield a meaningful amount of P(St-*alt*-PFS) copolymers, yet maintaining comonomer conversion ≤10 mol% to satisfy the Fineman-Ross method. The copolymerization solution was diluted with THF (1 mL) and then precipitated in ethanol (10 mL). After centrifugation, the precipitate was collected and dried in vacuum at 40 °C.

### General characterization

^1^H NMR and ^19^F NMR spectroscopy was conducted on a Varian Mercury plus 500 MHz spectrometer and a Bruker AV 400 MHz spectrometer using CDCl_3_ as the solvent. Chemical shifts were obtained using solvent residue as the reference. TEM was conducted on a JEM-1400 plus Microscope (Japan) at 120 kV. High-resolution TEM was conducted on a JEOL JEM2011F Microscope (Japan) at 200 kV. TEM samples were prepared by dropping 10 μL of the ethanolic dispersions (0.05% w/v or 0.1% w/v) on carbon-coated copper grids, followed by vacuum drying at 40 °C for 24 h. GPC was performed on an Agilent 1260 Infinity module with the Waters 2410 refractive index detector. Calibration was obtained using a series of monodisperse polystyrenes (molecular weight rang 5.8 × 10^3^–8.53 × 10^5^ g mol^−1^) with THF as the mobile phase. The flow rate of THF was 1.0 mL min^−1^ at 35 °C. Field-emission SEM was performed on a Zeiss Ultra 55 at a working voltage of 20 kV. The dispersions were diluted to 0.05% or 0.1% w/v and dropped onto mica sheet which were then attached to steel stubs using carbon adhesive. The samples were made conductive by sputtering a thin layer of gold after drying under vacuum at 40 °C for 5 h. SAXS experiments were recorded on a France/Xenocs XeUSS2.0 with microfocus liquid metal anodes Ga alloy at 70 kV. The experiments were carried out through the radiation of X-ray with a wavelength of *λ* = 1.34 Å at room temperature (25 °C). To prepare the SAXS samples, 0.5 mL BCP dispersions (30% w/v) were dialysis against 2% toluene/ethanol for 12 h and dropped on glass slides and dried at room temperature for 12 h and then further dried under vacuum at 25 °C for 2 h.

## Supplementary information

Supplementary Information

## Data Availability

The data that support the findings of this study are available from the corresponding author upon reasonable request.
