# Supplementary material for: Scalable preparation of alternating block copolymer particles with inverse bicontinuous mesophases
Source: Nat Commun. 2019 Mar 27;10:1397. doi: 10.1038/s41467-019-09324-5 (PMC6437182; doi:10.1038/s41467-019-09324-5)
Supplement: Supplementary file 1 — Supplementary Information [file 41467_2019_9324_MOESM1_ESM.pdf]

Scalable Preparation of Alternating Block Copolymer Particles with Inverse Bicontinuous Mesophases

Lv et al.

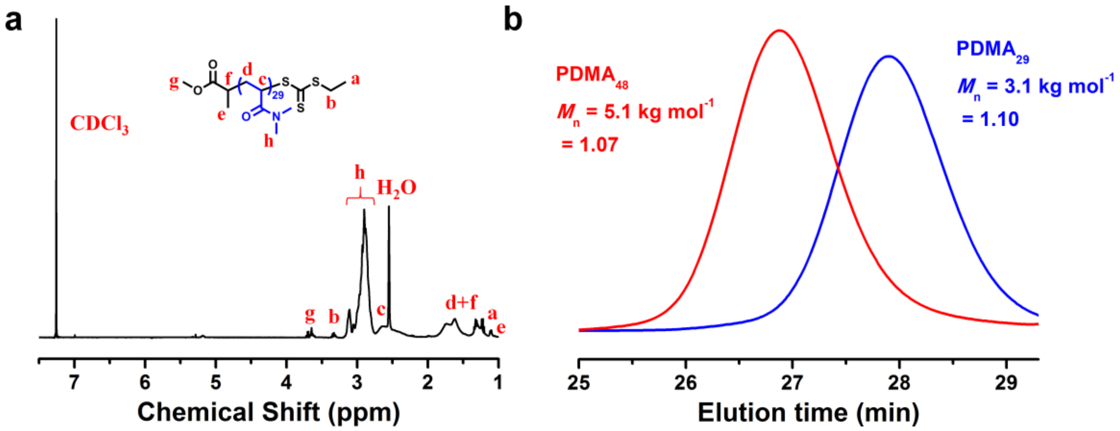

Supplementary Figure 1. Characterization of PDMA block. (**a**) Chemical structure and ^{1}H NMR spectrum of $PDMA_{29}$ in $CDCl_3$, (**b**) GPC traces of $PDMA_{29}$ and $PDMA_{48}$.

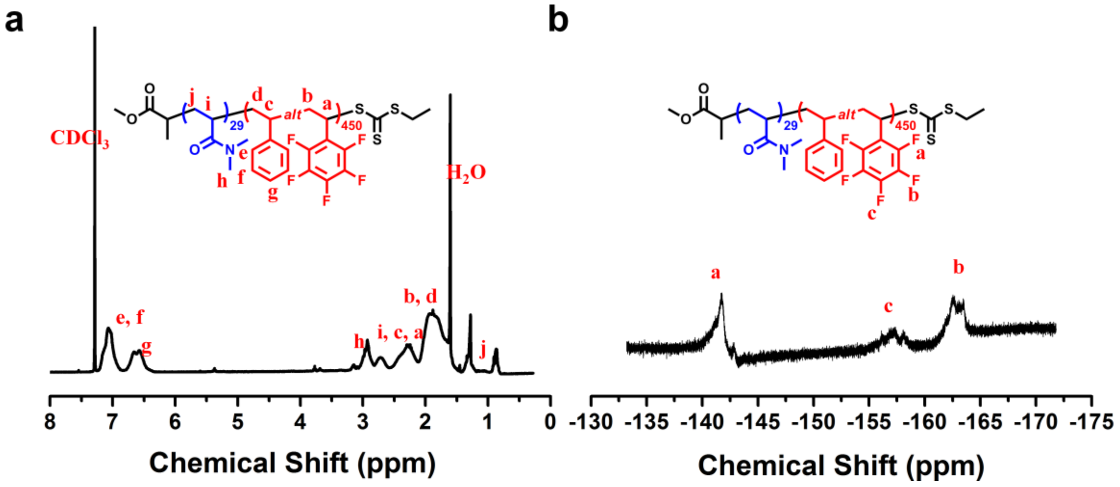

Supplementary Figure 2. Characterization of $PDMA_{29}$-*b*-P(St-*alt*-PFS)$_{450}$ copolymer. (**a**) Chemical structure and ^{1}H NMR spectrum of $PDMA_{29}$-*b*-P(St-*alt*-PFS)$_{450}$ in $CDCl_3$, (**b**) ^{19}F NMR spectrum of $PDMA_{29}$-*b*-P(St-*alt*-PFS)$_{450}$ in $CDCl_3$.

The Fineman-Ross equation:

$$G = r_1 F - r_2 \quad \text{Eq. (1)}$$

$$G = R - \frac{R}{\rho}, F = \frac{R^2}{\rho} \quad \text{Eq. (2)}$$

$$R = \frac{[M_1]}{[M_2]}, \rho = \frac{n1}{n2} = \frac{\frac{A(aryl-H)}{5}}{\frac{A(CH2-CH)}{3} - \frac{A(aryl-H)}{5}} \quad \text{Eq. (3)}$$

Supplementary Table 1. Values of variable calculated by Fineman-Ross equation. [a]

Entry	[St]:[PFS] [b]	[St]:[PFS] [c]	$M_{n,GPC}$ [d] (kg mol^{-1})	*Đ*	*R*	*ρ*	*G*	*F*
1	20:80	17.2:40.8	4.65	1.20	0.25	0.4216	-0.3430	0.1482
2	40:60	16.6:12.4	3.95	1.29	0.67	1.3387	0.1695	0.3353
3	50:50	17.6:10.4	4.60	1.26	1	1.6920	0.6134	1.3298
4	60:40	17.4:10.3	4.41	1.28	1.5	1.6886	0.6117	1.3325
5	80:20	18.4:6.27	3.79	1.30	4	2.9346	2.6370	5.4522

[a] All synthesis was conducted at 30% w/v solid content, 70 °C in ethanol,

[b] Feed ratio of St and PFS,

[c] Copolymer composition of St and PFS calculated from ^{1}H NMR spectroscopy analysis,

[d] Molecular weight determined by GPC (THF, PS).

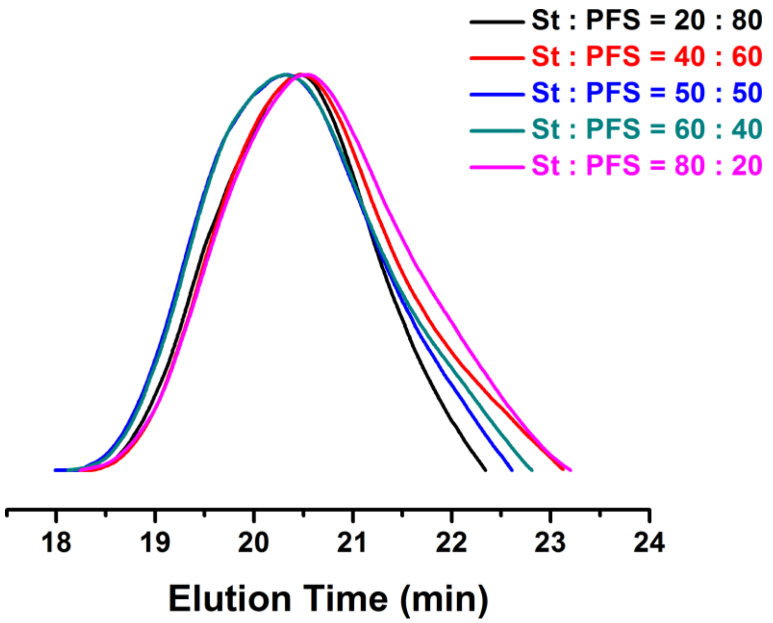

Supplementary Figure 3. GPC traces of P(St-*alt*-PFS) copolymers obtained for reactivity ratio determination.

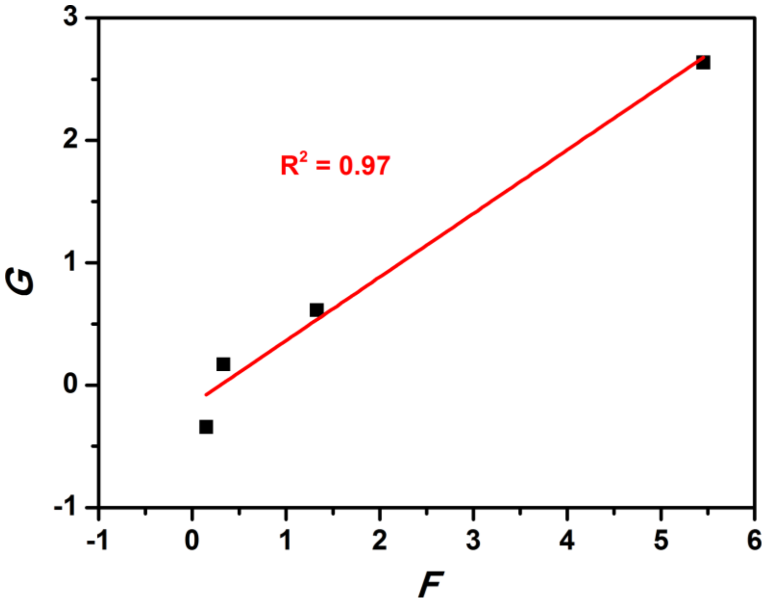

Supplementary Figure 4. Fineman-Ross plot used to determine the reactivity ratios of St and PFS in free radical copolymerization in ethanol.

Supplementary Table 2. Characterization data for PDMA29-*b*-P(St-*alt*-PFS)x copolymers synthesized in ethanol. [a]

Entry	Composition	Feed (St/PFS/PDMA29)	Composition (St/PFS)	DP (St+PFS)	Conv. [b] (%)	$M_{n,th}$ [c] (kg mol^{-1})	$M_{n,GPC}$ [d] (kg mol^{-1})	Đ [d]	Morphology
1	PDMA29-*b*-P(St-*alt*-PFS)89	50/50/1	45/45	89	89%	16.4	10.6	1.19	sphere
2	PDMA29-*b*-P(St-*alt*-PFS)108	60/60/1	54/54	108	90%	19.2	-	-	worm
3	PDMA29-*b*-P(St-*alt*-PFS)123	70/70/1	62/62	123	88%	21.7	18.3	1.17	jellyfish-like
4	PDMA29-*b*-P(St-*alt*-PFS)144	80/80/1	77/77	144	91%	24.6	18.5	1.20	worm and vesicle
5	PDMA29-*b*-P(St-*alt*-PFS)191	110/110/1	96/96	191	87%	31.6	-	-	vesicle
5	PDMA29-*b*-P(St-*alt*-PFS)237	130/130/1	119/119	237	91%	38.4	33.6	1.18	vesicle
6	PDMA29-*b*-P(St-*alt*-PFS)270	150/150/1	135/135	270	90%	43.4	34.5	1.19	LCV
7	PDMA29-*b*-P(St-*alt*-PFS)310	170/170/1	155/155	310	91%	49.3	-	-	LCV and sponge-like
9	PDMA29-*b*-P(St-*alt*-PFS)342	190/190/1	171/171	342	90%	54.1	-	-	LCV and spong-like
10	PDMA29-*b*-P(St-*alt*-PFS)356	200/200/1	178/178	356	89%	56.2	41.9	1.20	sponge-like
11	PDMA29-*b*-P(St-*alt*-PFS)450	250/250/1	225/225	450	90%	70.2	60.3	1.21	cubosome
12	PDMA29-*b*-P(St-*alt*-PFS)552	300/300/1	276/276	552	92%	85.4	-	-	cubosome

[a] All copolymers synthesized at 30% w/v solid content, 70 °C in ethanol,

[b] Monomer conversion determined by ^{1}H NMR spectroscopy analysis,

[c] Theoretical molecular weight of copolymers = target DP_{St} × St conversion × M_{St} + target DP_{PFS} × PFS conversion + 29 × M_{DMA} + M_{CTA},

[d] Molecular weight determined by GPC (THF, PS).

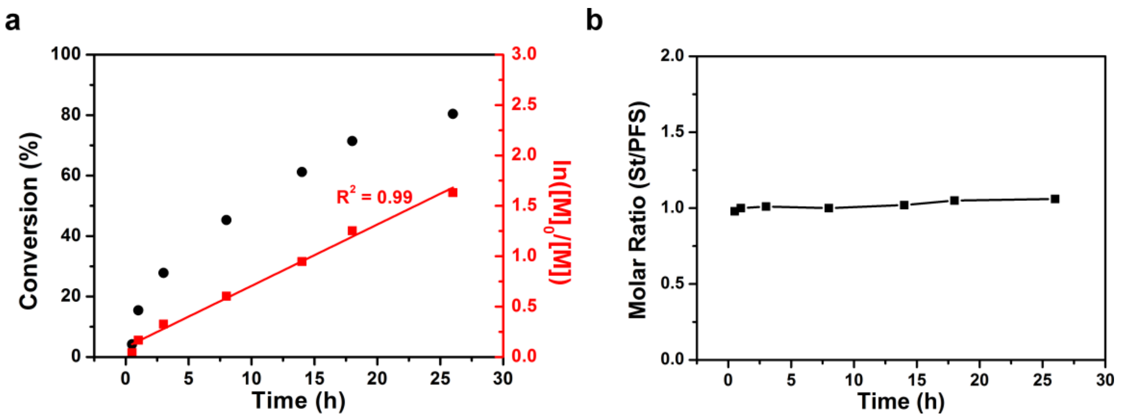

Supplementary Figure 5. Research for kinetics of RAFT copolymerization of St and PFS. (**a**) Kinetic plots for the RAFT ethanolic dispersion polymerization of St and PFS ([PDMA29]/[St]/[PFS]/[AIBN] = 1/250/250/0.3, 30% w/v solid content, 70 °C). (**b**) Molar ratio of remaining [St]/[PFS] at different time.

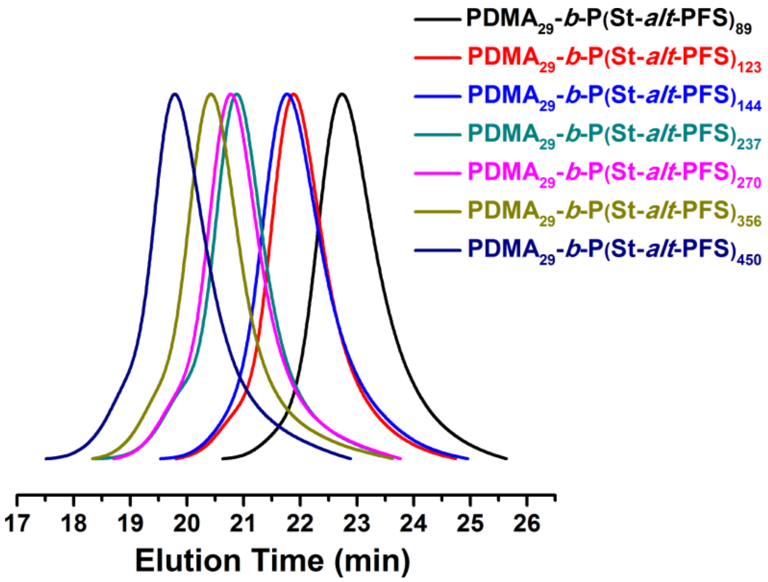

Supplementary Figure 6. GPC traces of PDMA$_{29}$-*b*-P(St-*alt*-PFS)$_x$ copolymers synthesized in ethanol at 30% w/v solid content, 70 °C.

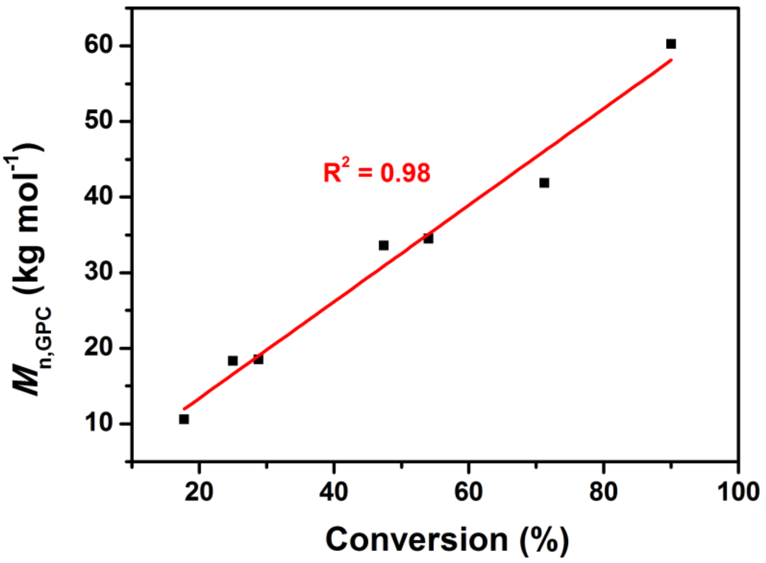

Supplementary Figure 7. Dependence of $M_{n,GPC}$ on the monomer conversion for PDMA$_{29}$-*b*-P(St-*alt*-PFS)$_x$ copolymers synthesized in ethanol at 30% w/v solid content, 70 °C.

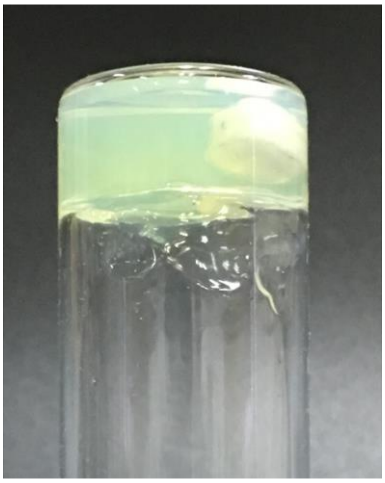

Supplementary Figure 8. Digital photograph of PDMA$_{29}$-*b*-P(St-*alt*-PFS)$_{108}$ BCP dispersion synthesized in ethanol at 30% w/v solid content, 70 °C.

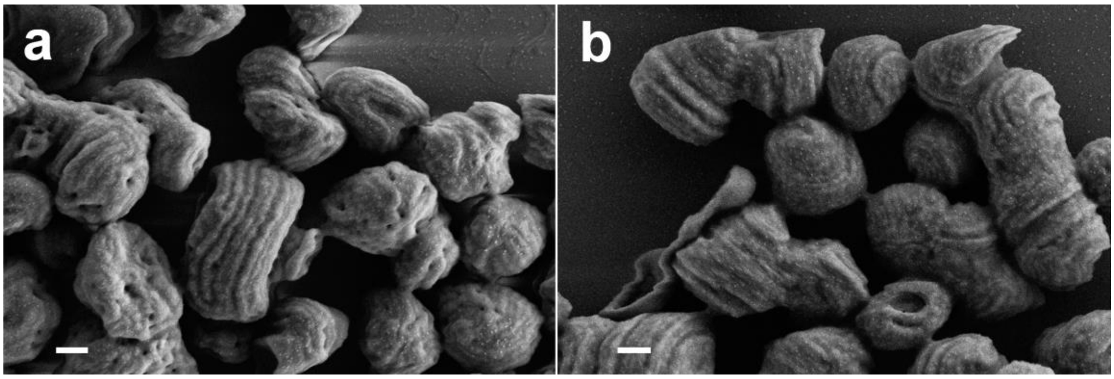

Supplementary Figure 9. SEM micrographs for two particles synthesized in ethanol at 30% w/v solid content, 70 °C. (**a**) PDMA$_{29}$-*b*-P(St-*alt*-PFS)$_{356}$, (**b**) PDMA$_{29}$-*b*-P(St-*alt*-PFS)$_{450}$ (All scale bars: 300 nm).

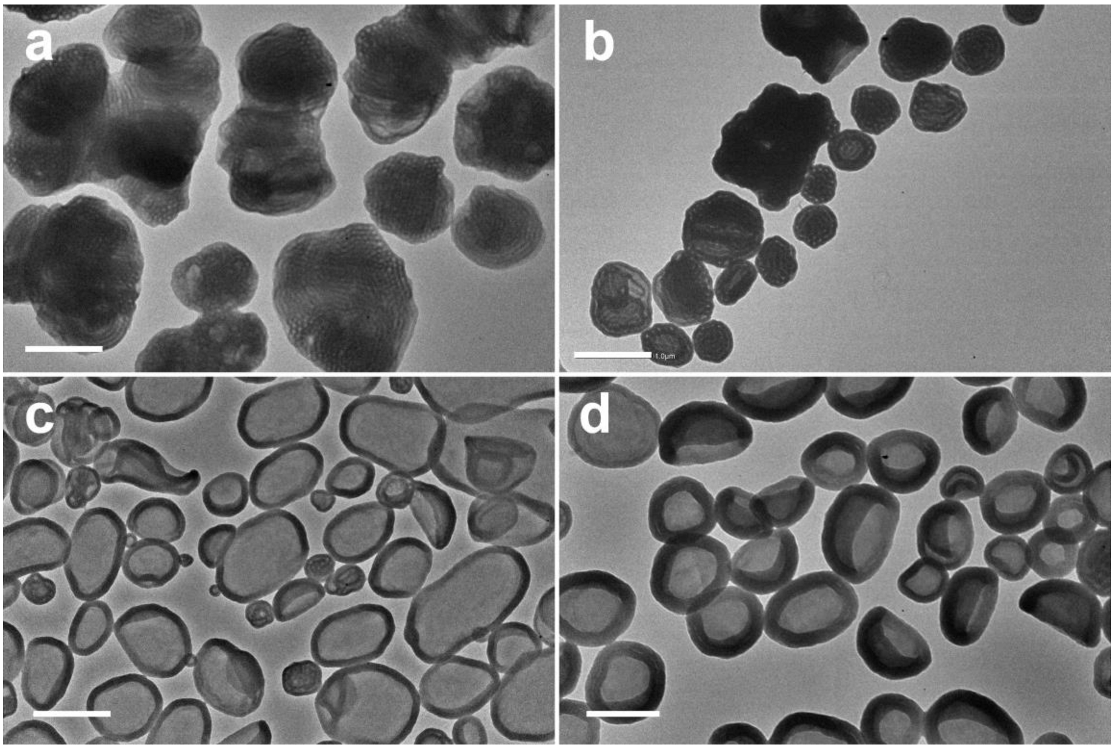

Supplementary Figure 10. TEM micrographs for particles (target DP 500) synthesized at different solid contents in ethanol, 70 °C. (**a**) 40% w/v, $PDMA_{29}$-*b*-P(St-*alt*-PFS)$_{465}$ (Scale bar: 1 μm), (**b**) 35% w/v, $PDMA_{29}$-*b*-P(St-*alt*-PFS)$_{450}$ (Scale bar: 1 μm), (**c**) 25% w/v, $PDMA_{29}$-*b*-P(St-*alt*-PFS)$_{450}$ (Scale bar: 1 μm), (d) 20% w/v, $PDMA_{29}$-*b*-P(St-*alt*-PFS)$_{440}$ (Scale bar: 500 nm).

Supplementary Table 3. Characterization data for $PDMA_{29}$-*b*-P(St-*alt*-PFS)$_{x}$ copolymers (target DP 500) synthesized at different solid contents. [a]

Entry	Composition	Feed (St/PFS/$PDMA_{29}$)	Solid Content %	Composition (St/PFS)	DP (St+PFS)	Conv. [b] (%)	Morphology
1	$PDMA_{29}$-*b*-P(St-*alt*-PFS)$_{465}$	250/250/1	40	233/233	465	93	cubosome
2	$PDMA_{29}$-*b*-P(St-*alt*-PFS)$_{450}$		35	225/225	450	90	sponge-like
3	$PDMA_{29}$-*b*-P(St-*alt*-PFS)$_{450}$		25	225/225	450	90	(elongated) vesicle
4	$PDMA_{29}$-*b*-P(St-*alt*-PFS)$_{440}$		20	220/220	440	88	vesicle

[a] All copolymers synthesized at 70 °C, in ethanol,

[b] Monomer conversion determined by ^{1}H NMR spectroscopy analysis,

[c] Theoretical molecular weight of copolymers = target DP_{St} × St conversion × M_{St} + target DP_{PFS} × PFS conversion + 29 × M_{DMA} + M_{CTA}.

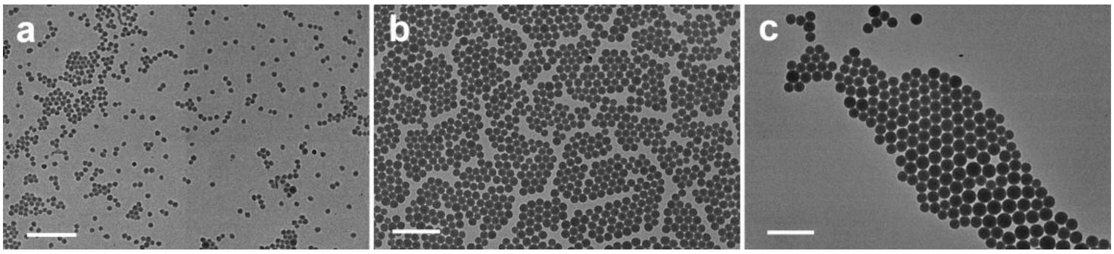

Supplementary Figure 11. TEM images for particles synthesized via ethanolic dispersion copolymerization using PDMA$_{48}$ at 30% w/v solid content, 70 °C. (**a**) PDMA$_{48}$-*b*-P(St-*alt*-PFS)$_{218}$ (Scale bar: 200 nm), (**b**) PDMA$_{48}$-*b*-P(St-*alt*-PFS)$_{540}$ (Scale bar: 500 nm), (**c**) PDMA$_{48}$-*b*-P(St-*alt*-PFS)$_{880}$ (Scale bar: 500 nm).

Supplementary Table 4. Characterization data for PDMA$_{48}$-*b*-P(St-*alt*-PFS)$_x$ copolymers. [a]

Entry	Composition	Feed (St/PFS/PDMA$_{48}$)	Composition (St/PFS)	DP (St+PFS)	Conv. [b] (%)	D_n [c] (nm)	Morphology
1	PDMA$_{48}$-*b*-P(St-*alt*-PFS)$_{218}$	125/125/1	109/109	218	87	36.4 ± 4.8	sphere
2	PDMA$_{48}$-*b*-P(St-*alt*-PFS)$_{540}$	300/300/1	270/270	540	90	82.3 ± 6.5	sphere
3	PDMA$_{48}$-*b*-P(St-*alt*-PFS)$_{880}$	500/500/1	440/440	880	88	145.8 ± 10.1	sphere

[a] All copolymers synthesized at 30% w/v solid Content, 70 °C in ethanol,
[b] Monomer conversion determined by ^{1}H NMR spectroscopy analysis,
[c] Number-mean particle diameter determined by TEM analysis.

Supplementary Table 5. Characterization data for PDMA$_{29}$-*b*-P(St-*alt*-PFS)$_x$ copolymers (target DP 500) synthesized using different cosolvents. [a]

Entry	Composition	Feed (St/PFS/PDMA$_{29}$)	Composition (St/PFS)	Solvent	DP (St+PFS)	Conv. [b] (%)	Morphology
1	PDMA$_{29}$-*b*-P(St-*alt*-PFS)$_{465}$	250/250/1	233/233	2% toluene + 98% EtOH	465	93	cubosome
2	PDMA$_{29}$-*b*-P(St-*alt*-PFS)$_{455}$		228/228	2% THF + 98% EtOH	455	91	cubosome+sponge
3	PDMA$_{29}$-*b*-P(St-*alt*-PFS)$_{405}$		203/203	2% DMF + 98% EtOH	405	81	cubosome
4	PDMA$_{29}$-*b*-P(St-*alt*-PFS)$_{450}$		225/225	2% 1,4-diaxone + 98% EtOH	450	90	sponge

[a] All copolymers synthesized at 30% w/v solid content, 70 °C,
[b] Monomer conversion determined by ^{1}H NMR spectroscopy analysis.

Supplementary Table 6. Characterization data for PDMA$_{29}$-*b*-P(St-*alt*-PFS)$_x$ copolymers synthesized in toluene/ethanol (2/98, v/v). [a]

Entry	Composition	Feed (St/PFS/PDMA$_{29}$)	Composition (St/PFS)	DP (St+PFS)	Conv. [b] (%)	$M_{n,th}$ [c] (kg mol^{-1})	$M_{n,GPC}$ [d] (kg mol^{-1})	$Đ$ [d]	Morphology
1	PDMA$_{29}$-*b*-P(St-*alt*-PFS)$_{97}$	50/50/1	49/49	97	97	17.6	13.8	1.25	sphere
2	PDMA$_{29}$-*b*-P(St-*alt*-PFS)$_{129}$	65/65/1	65/65	129	99	22.3	19.4	1.21	worm
3	PDMA$_{29}$-*b*-P(St-*alt*-PFS)$_{198}$	100/100/1	99/99	198	99	32.6	30.2	1.17	vesicle
4	PDMA$_{29}$-*b*-P(St-*alt*-PFS)$_{294}$	150/150/1	147/147	294	98	46.9	40.3	1.20	vesicle
5	PDMA$_{29}$-*b*-P(St-*alt*-PFS)$_{388}$	200/200/1	194/194	388	97	61.0	57.9	1.22	elongated vesicle+LCV +sponge
6	PDMA$_{29}$-*b*-P(St-*alt*-PFS)$_{428}$	225/225/1	214/214	428	95	66.9	62.1	1.19	cubosome
7	PDMA$_{29}$-*b*-P(St-*alt*-PFS)$_{490}$	250/250/1	245/245	490	98	76.2	58.4	1.19	cubosome
8	PDMA$_{29}$-*b*-P(St-*alt*-PFS)$_{544}$	275/275/1	272/272	544	99	84.2	82.9	1.30	cubosome+ hexasome
9	PDMA$_{29}$-*b*-P(St-*alt*-PFS)$_{582}$	300/300/1	291/291	582	97	89.9	89.1	1.29	cubosome+ hexasome
10	PDMA$_{29}$-*b*-P(St-*alt*-PFS)$_{637}$	325/325/1	319/319	637	98	98.1	92.3	1.34	cubosome+ hexasome

[a] All copolymers synthesized at 30% w/v solid content, 70 °C, in toluene/ethanol (2/98, v/v),

[b] Monomer conversion determined by ^{1}H NMR spectroscopy analysis,

[c] Theoretical molecular weight of copolymers = (target DP$_{(PFS+St)}$ × monomer conversion) / 2 × (M_{PFS}+ M_{St}) + 29 × M_{DMA} + M_{CTA},

[d] Molecular weight determined by GPC (THF, PS).

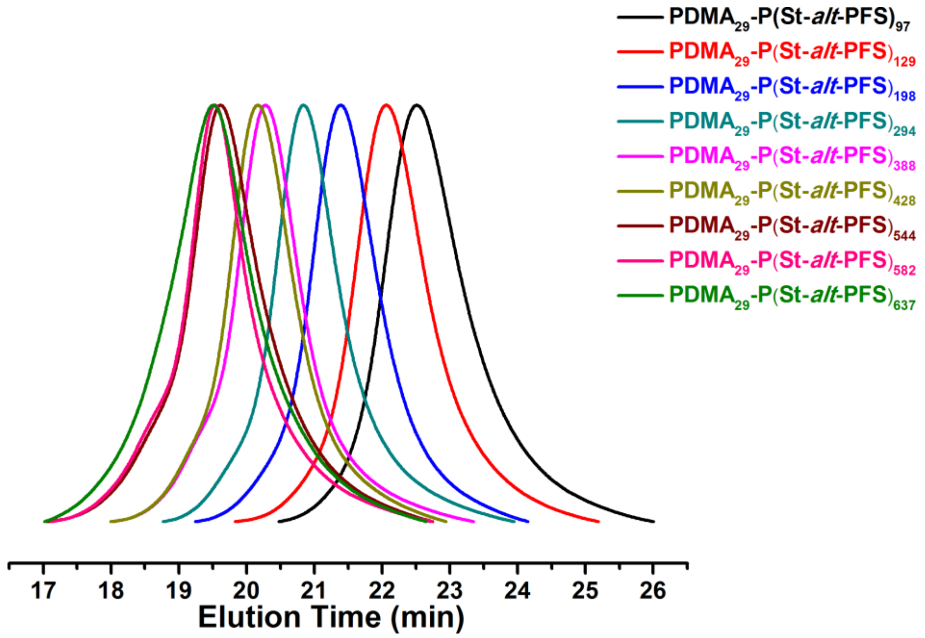

Supplementary Figure 12. GPC traces of PDMA$_{29}$-*b*-P(St-*alt*-PFS)$_x$ copolymers synthesized in toluene/ethanol (2/98, v/v) at 30% w/v solid content, 70 °C.

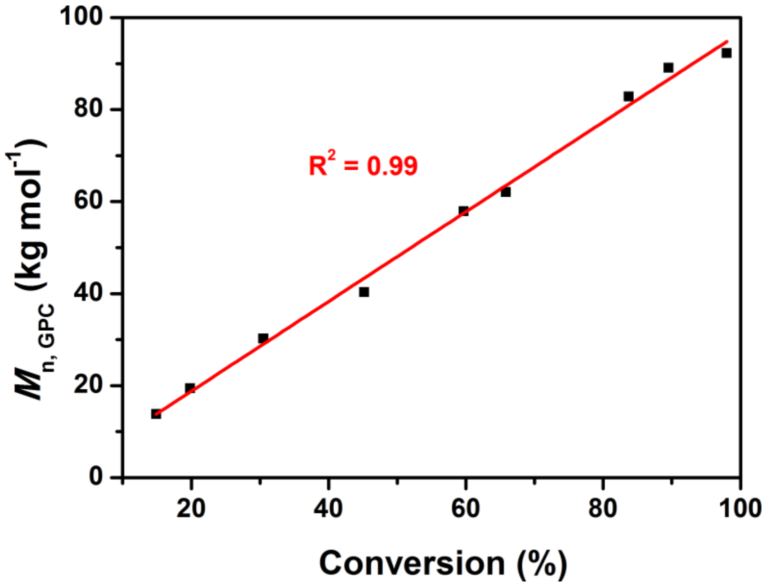

Supplementary Figure 13. Dependence of $M_{n,GPC}$ on the monomer conversion for PDMA$_{29}$-*b*-P(St-*alt*-PFS)$_x$ BCPs synthesized in toluene/ethanol (2/98, v/v) at 30% w/v solid content, 70 °C.

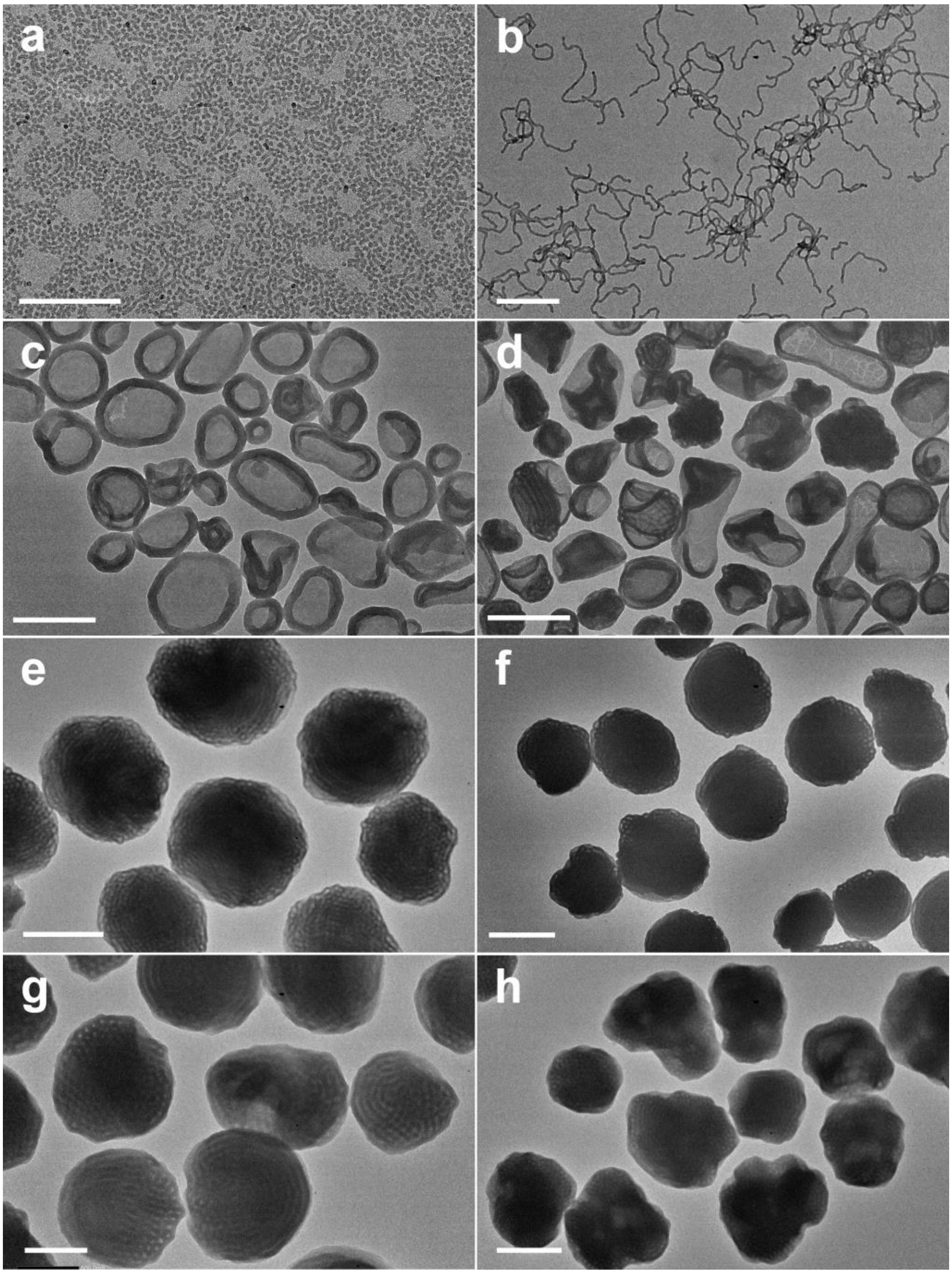

Supplementary Figure 14. TEM micrographs for particles synthesized at 30% w/v solid content, 70 °C in toluene/ethanol (2/98, v/v). (**a**) $PDMA_{29}$-*b*-P(St-*alt*-PFS)$_{97}$ (Scale bar: 500 nm), (**b**) $PDMA_{29}$-*b*-P(St-*alt*-PFS)$_{129}$ (Scale bar: 500 nm), (**c**) $PDMA_{29}$-*b*-P(St-*alt*-PFS)$_{294}$ (Scale bar: 500 nm), (**d**) $PDMA_{29}$-*b*-P(St-*alt*-PFS)$_{388}$ (Scale bar: 1 μm), (**e**) $PDMA_{29}$-*b*-P(St-*alt*-PFS)$_{428}$ (Scale bar: 500 nm), (**f**) $PDMA_{29}$-*b*-P(St-*alt*-PFS)$_{490}$ (Scale bar: 1 μm), (**g**) $PDMA_{29}$-*b*-P(St-*alt*-PFS)$_{582}$ (Scale bar: 500 nm), (**h**) $PDMA_{29}$-*b*-P(St-*alt*-PFS)$_{637}$ (Scale bar: 1 μm).

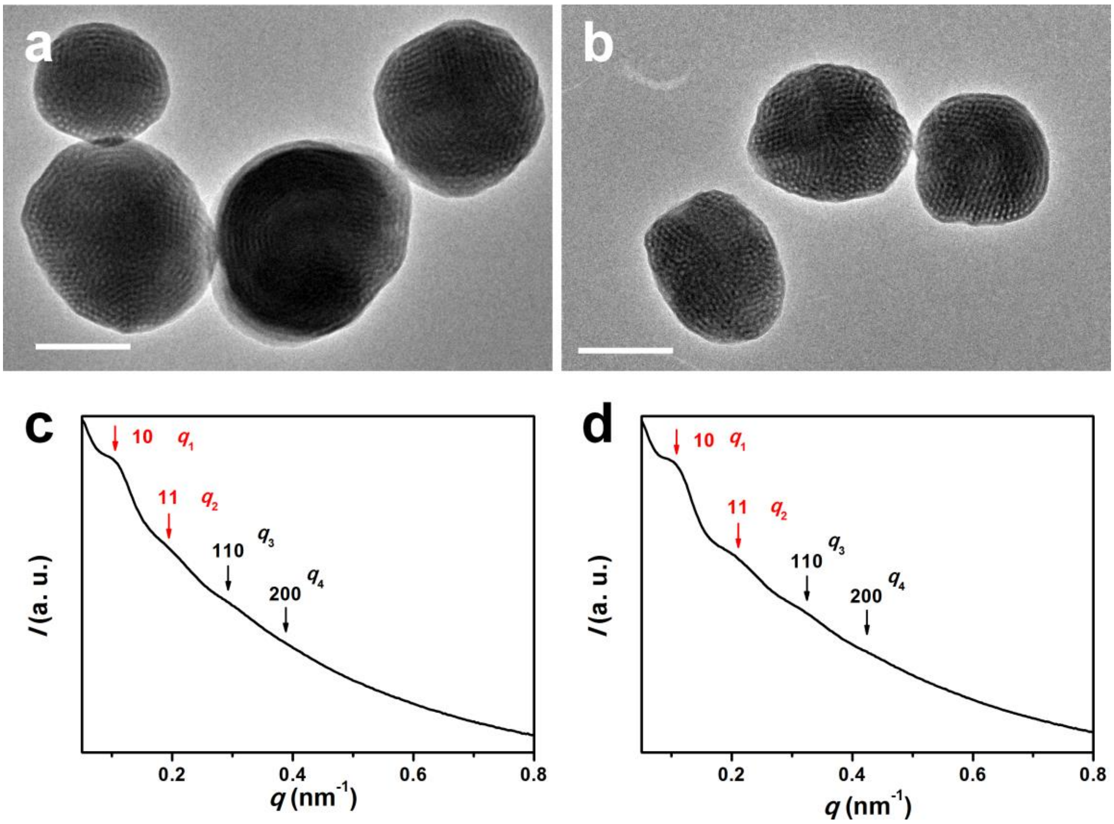

Supplementary Figure 15. TEM micrographs and SAXS patterns for particles synthesized at targeting PDMA$_{29}$-*b*-P(St-*alt*-PFS)$_{600}$ at 70 °C in toluene/ethanol (2/98, v/v). (**a**) and (**c**) 35% w/v solid content, (**b**) and (**d**) 40% solid content (All scale bars: 1 μm).

Supplementary Table 7. Characterization data for PDMA$_{29}$-*b*-P(St-*alt*-PFS)$_x$ copolymers synthesized in toluene/ethanol (5/95, v/v). [a]

Entry	Composition	Feed (St/PFS/PDMA$_{29}$)	Composition (St/PFS)	DP (St+PFS)	Conv. [b] (%)	$M_{n,th}$ [c] (kg mol^{-1})	$M_{n,GPC}$ [d] (kg mol^{-1})	*Đ* [d]	Morphology
1	PDMA$_{29}$-*b*-P(St-*alt*-PFS)$_{98}$	50/50/1	49/49	98	98	17.7	13.2	1.24	sphere
2	PDMA$_{29}$-*b*-P(St-*alt*-PFS)$_{194}$	100/100/1	97/97	194	97	32.0	25.6	1.23	vesicle
3	PDMA$_{29}$-*b*-P(St-*alt*-PFS)$_{285}$	150/150/1	143/143	285	95	45.6	38.1	1.20	LCV
4	PDMA$_{29}$-*b*-P(St-*alt*-PFS)$_{364}$	200/200/1	182/182	364	91	57.4	50.3	1.22	sponge
5	PDMA$_{29}$-*b*-P(St-*alt*-PFS)$_{400}$	250/250/1	200/200	400	80	62.7	51.0	1.20	sponge+cubosome
6	PDMA$_{29}$-*b*-P(St-*alt*-PFS)$_{564}$	300/300/1	282/282	564	94	87.2	74.7	1.31	cubosome

[a] All copolymers synthesized at 30% w/v solid content, 70 °C, in toluene/ethanol (5/95, v/v),

[b] Monomer conversion determined by ^{1}H NMR spectroscopy analysis,

[c] Theoretical molecular weight of copolymers = (target DP$_{(PFS+St)}$ × monomer conversion) / 2 × (M_{PFS}+ M_{St}) + 29 × M_{DMA} + M_{CTA},

[d] Molecular weight determined by GPC (THF, PS).

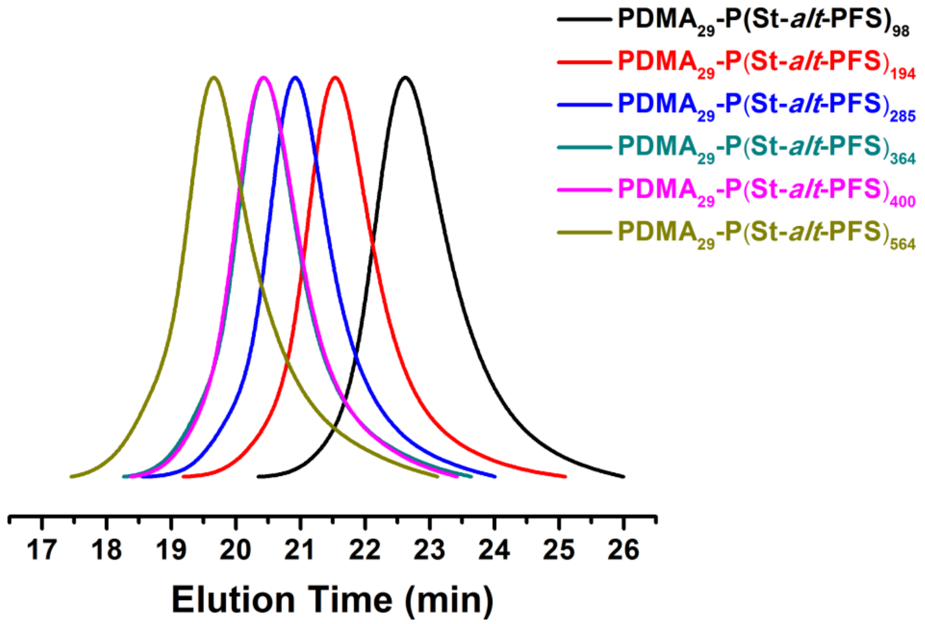

Supplementary Figure 16. GPC traces of $PDMA_{29}$-*b*-P(St-*alt*-PFS)$_x$ copolymers synthesized in toluene/ethanol (5/95, v/v) at 30% w/v solid content, 70 °C.

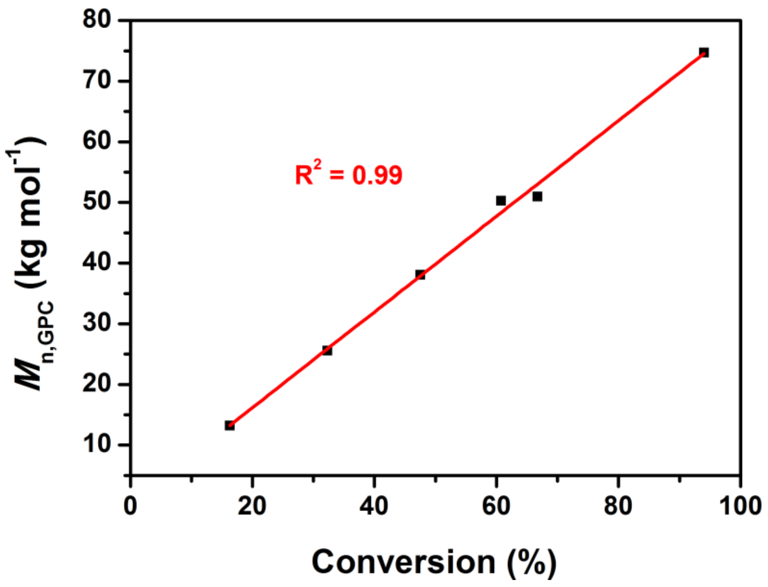

Supplementary Figure 17. Dependence of $M_{n,GPC}$ on the monomer conversion for $PDMA_{29}$-*b*-P(St-*alt*-PFS)$_x$ copolymers synthesized in toluene/ethanol (5/95, v/v) at 30% w/v solid content, 70 °C.

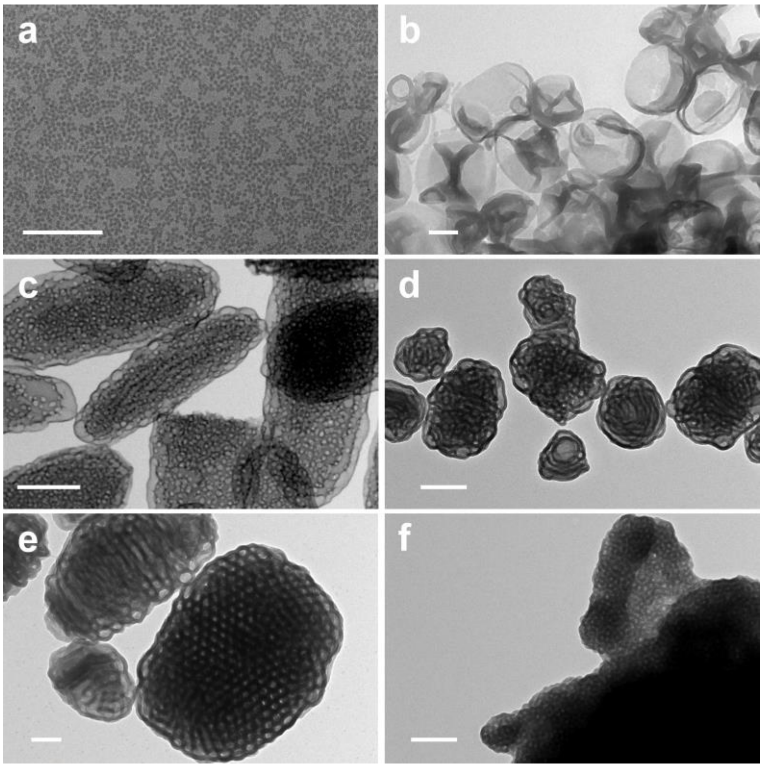

Supplementary Figure 18. TEM micrographs for **particles synthesized in toluene/ethanol (5/95, v/v) at 30% w/v solid content, 70 °C.** (**a**) $PDMA_{29}$-*b*-P(St-*alt*-PFS)$_{98}$ (Scale bar: 500 nm), (**b**) $PDMA_{29}$-*b*-P(St-*alt*-PFS)$_{194}$ (Scale bar: 200 nm), (**c**) $PDMA_{29}$-*b*-P(St-*alt*-PFS)$_{285}$ (Scale bar: 1 µm), (**d**) $PDMA_{29}$-*b*-P(St-*alt*-PFS)$_{364}$ (Scale bar: 500 nm)**,** (**e**) $PDMA_{29}$-*b*-P(St-*alt*-PFS)$_{400}$ (Scale bar: 200 nm), (**f**) $PDMA_{29}$-*b*-P(St-*alt*-PFS)$_{564}$ (Scale bar: 500 nm).

Supplementary Table 8. Characterization data for PDMA29-*b*-P(St-*alt*-PFS)x copolymers synthesized in toluene/ethanol (10/90, v/v). [a]

Entry	Composition	Feed (St/PFS/PDMA29)	Composition (St/PFS)	DP (St+PFS)	Conv. [b] (%)	$M_{n,th}$ [c] (kg mol^{-1})	$M_{n,GPC}$ [d] (kg mol^{-1})	Đ [d]	Morphology
1	PDMA29-*b*-P(St-*alt*-PFS)94	50/50/1	47/47	94	94	17.1	12.7	1.20	sphere
2	PDMA29-*b*-P(St-*alt*-PFS)184	100/100/1	92/92	184	92	30.5	25.0	1.20	vesicle
3	PDMA29-*b*-P(St-*alt*-PFS)344	200/200/1	172/172	344	86	54.4	42.0	1.21	LCV
4	PDMA29-*b*-P(St-*alt*-PFS)355	250/250/1	178/178	355	71	56.0	43.9	1.22	sponge
5	PDMA29-*b*-P(St-*alt*-PFS)362	300/300/1	181/181	362	60	57.1	45.8	1.27	sponge

[a] All copolymers synthesized at 30% w/v solid content, 70 °C, in toluene/ ethanol (10/90, v/v),

[b] Monomer conversion determined by ^{1}H NMR spectroscopy analysis,

[c] Theoretical molecular weight of copolymers = (target DP$_{(PFS+St)}$ × monomer conversion) / 2 × (M_{PFS}+ M_{St}) + 29 × M_{DMA} + M_{CTA},

[d] Molecular weight determined by GPC (THF, PS).

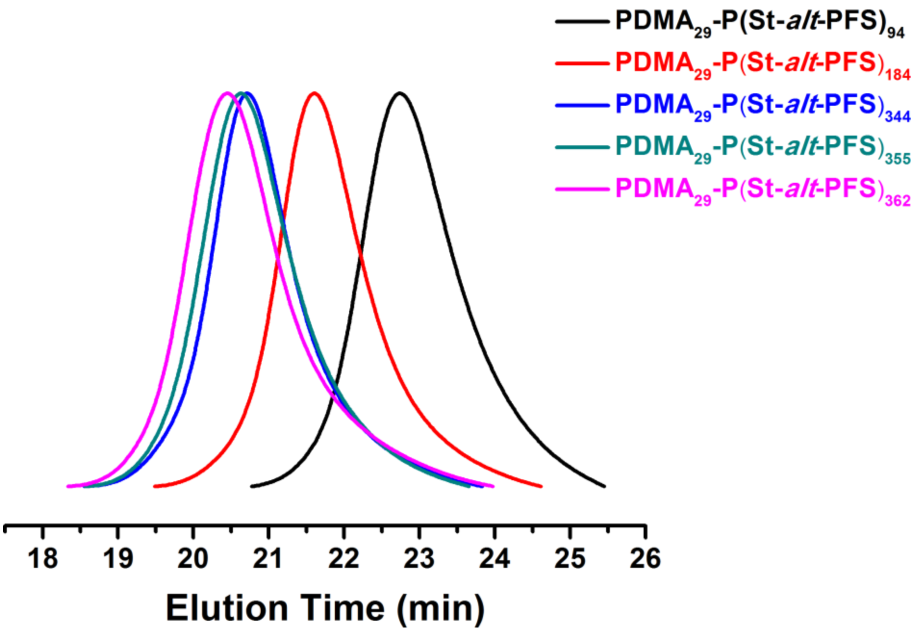

Supplementary Figure 19. GPC traces of PDMA29-*b*-P(St-*alt*-PFS)x copolymers synthesized in toluene/ethanol (10/90, v/v) at 30% w/v solid content, 70 °C.

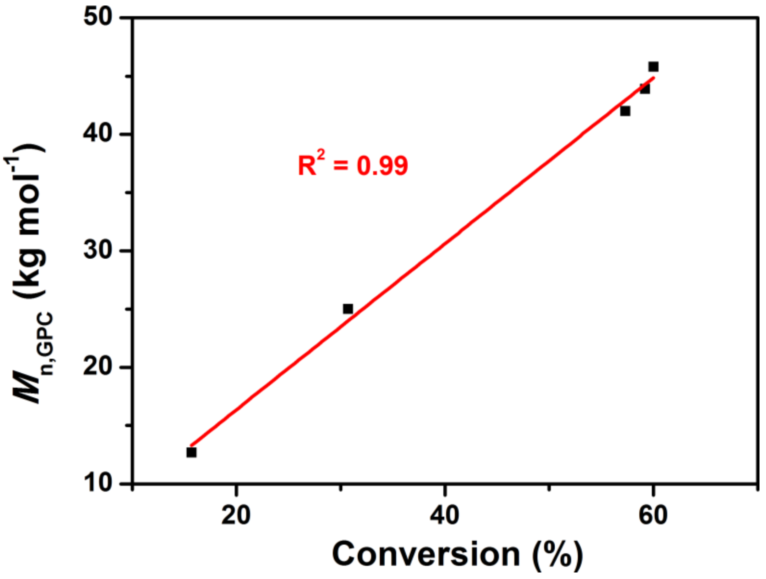

Supplementary Figure 20. Dependence of $M_{n,GPC}$ on the monomer conversion for PDMA$_{29}$-*b*-P(St-*alt*-PFS)$_x$ BCPs synthesized in toluene/ethanol (10/90, v/v) at 30% w/v solid content, 70 °C.

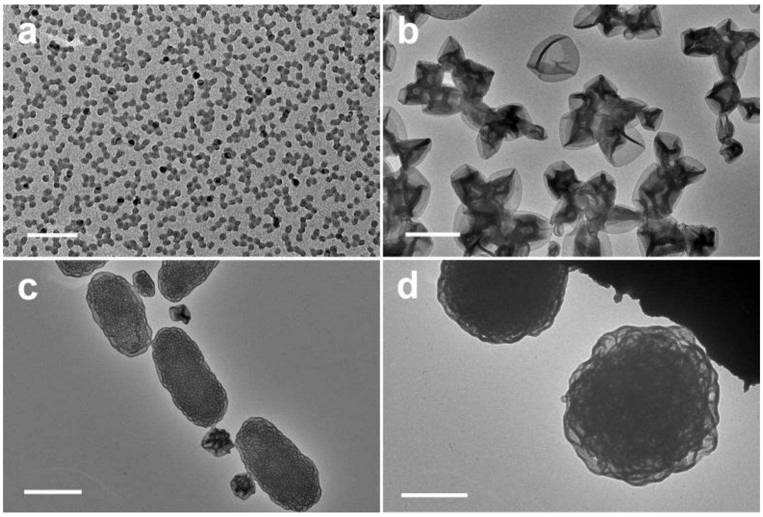

Supplementary Figure 21. TEM micrographs for particles synthesized at 30% w/v solid content, 70 °C in toluene/ethanol (10/90, v/v). (**a**) PDMA$_{29}$-*b*-P(St-*alt*-PFS)$_{94}$ (Scale bar: 200 nm), (**b**) PDMA$_{29}$-*b*-P(St-*alt*-PFS)$_{184}$ (Scale bar: 1 μm), (**c**) PDMA$_{29}$-*b*-P(St-*alt*-PFS)$_{344}$ (Scale bar: 2 μm), (**d**) PDMA$_{29}$-*b*-P(St-*alt*-PFS)$_{355}$ (Scale bar: 1 μm).
